# Plasma Lipidome Is Dysregulated in Amyotrophic Lateral Sclerosis and Associated With Life‐Course Adiposity Polygenic Risk

**DOI:** 10.1002/jcsm.70410

**Published:** 2026-10-21

**Authors:** Linjing Zhang, Leyuan Zhang, Yongping Zheng, Zhenhuang Zhuang, Lu Tang, Wei Zheng, Kailin Xia, Shan Ye, Tielun Yin, Tao Huang, Dongsheng Fan

**Affiliations:** ^1^ Department of Neurology Peking University Third Hospital Beijing China; ^2^ Institute of Genetics and Developmental Biology Chinese Academy of Sciences Beijing China; ^3^ University of Chinese Academy of Sciences Beijing China; ^4^ Department of Radiation Oncology Peking University Third Hospital Beijing China; ^5^ Department of Cardiology and Institute of Vascular Medicine Peking University Third Hospital Beijing China; ^6^ Department of Nutrition and Food Hygiene, School of Public Health Peking University Beijing China; ^7^ State Key Laboratory of General Artificial Intelligence Peking University Beijing China; ^8^ Beijing Key Lab of Innovative Diagnostics, Therapeutics and Translational Research for Neurological Rare Disorders Beijing China; ^9^ Key Laboratory for Neuroscience, National Health Commission/Ministry of Education Peking University Beijing China

**Keywords:** adiposity, amyotrophic lateral sclerosis, gene–environment interaction, lipidomic, polygenic risk score

## Abstract

**Objective:**

Although both pre‐symptomatic adiposity and dysregulated lipid metabolism are implicated in amyotrophic lateral sclerosis (ALS), the mechanistic pathways linking adiposity to disease remain unclear. This study aimed to investigate how genetic predisposition to adiposity might influence ALS through specific lipid metabolic pathways.

**Methods:**

We conducted mass spectrometry–based quantitative lipidomics on plasma from 104 ALS patients and 78 age‐ and sex‐matched controls. Life‐course adiposity polygenic risk scores (PRS) for birth weight, body fat percentage, appendicular lean mass and whole‐body fat‐free mass were constructed. Associations and PRS‐by‐disease interaction effects on the lipidome were evaluated.

**Results:**

Compared to controls, ALS patients exhibited widespread lipid dysregulation. Seventy‐eight lipid species were significantly altered (FDR  < 0.05, |Cohen's *d*| > 1.0), characterized by a predominant decrease in acylcarnitines (75% of species) and distinct shifts within sphingolipid and glycerophospholipid subclasses. A three‐acylcarnitine candidate panel (C10:0, C12:0 and C18:2) using fivefold analysis with feature selection repeated within each training fold yielded an overall out‐of‐fold AUC of 0.958. Exploratory PRS‐by‐disease‐status analyses identified 16 nominal interaction signals, including 13 acylcarnitines; no significant mediation effect of lipids between PRS and ALS risk was detected.

**Conclusion:**

These findings identify robust ALS‐associated plasma lipid alterations, and our exploratory findings suggest that genetic predisposition to adiposity may shape the pattern of lipid metabolic disruption in ALS, offering new insights into the disease's metabolic pathophysiology.

## 1 Introduction

Amyotrophic lateral sclerosis (ALS) is a fatal neurodegenerative disorder characterized by the progressive loss of motor neurons. Accumulating evidence indicates systemic metabolic alterations, including decrease of body mass index (BMI) [1], energy homeostasis [2], hypermetabolism [3, 4] and dysregulated lipid metabolism [5], are prominent features of this disease. BMI and adiposity have long been proposed to modify ALS risk and be predictive of survival [1, 5, 6, 7]. Body compositions, such as fat mass [5, 8, 9], BFP [10] or fat‐free mass or muscle mass [11], are also explored in several ALS studies, suggesting that these may serve as predictive factors for prognosis [8, 12]. On the other hand, lipid dysregulation is also a well‐known feature of MNDs [2], which has been genetically and potentially causally linked to disease risk [13, 14]; also, circulating lipid and metabolomic profiles have been associated with ALS prognosis [15, 16, 17].

Given that early life adiposity and body fat distribution are intrinsically linked to systemic lipid metabolism, individuals are consequently predisposed to adult disease [18, 19], it remains unknown whether genetic predisposition to adiposity interacts with the lipidome to influence ALS pathogenesis. And if yes, how genetic adiposity risk influences the lipidome in the context of ALS? This gap fundamentally limits our understanding of the roles of adipose tissue and lipid metabolism in ALS pathogenesis, thereby hindering the development of targeted therapies.

To address this, we firstly performed quantitative lipidomic profiling using ultraperformance liquid chromatography–tandem mass spectrometry (UPLC‐MS/MS) on matched plasma samples from ALS patients and controls and then constructed individual adiposity‐related PRS for these ALS patients and controls. Our aims were twofold: (1) to establish an integrated profile of core lipidomic perturbations in ALS and to develop and internally evaluate a machine learning–based candidate diagnostic model capturing these metabolic features and (2) to employ life‐course adiposity polygenic risk scores (PRS) for birth weight (BW), body fat percentage (BFP), appendicular lean mass (ALM) and whole‐body fat‐free mass (WBFFM) to explore, via mediation and interaction models, how genetic adiposity risk influences the lipidome in the context of ALS. We anticipate that this study will provide a foundational framework for elucidating the complex, multifaceted roles of body composition and lipid metabolism in ALS pathophysiology.

## 2 Materials and Methods

The overall study flow was drawn in Figure 1.

**FIGURE 1:**
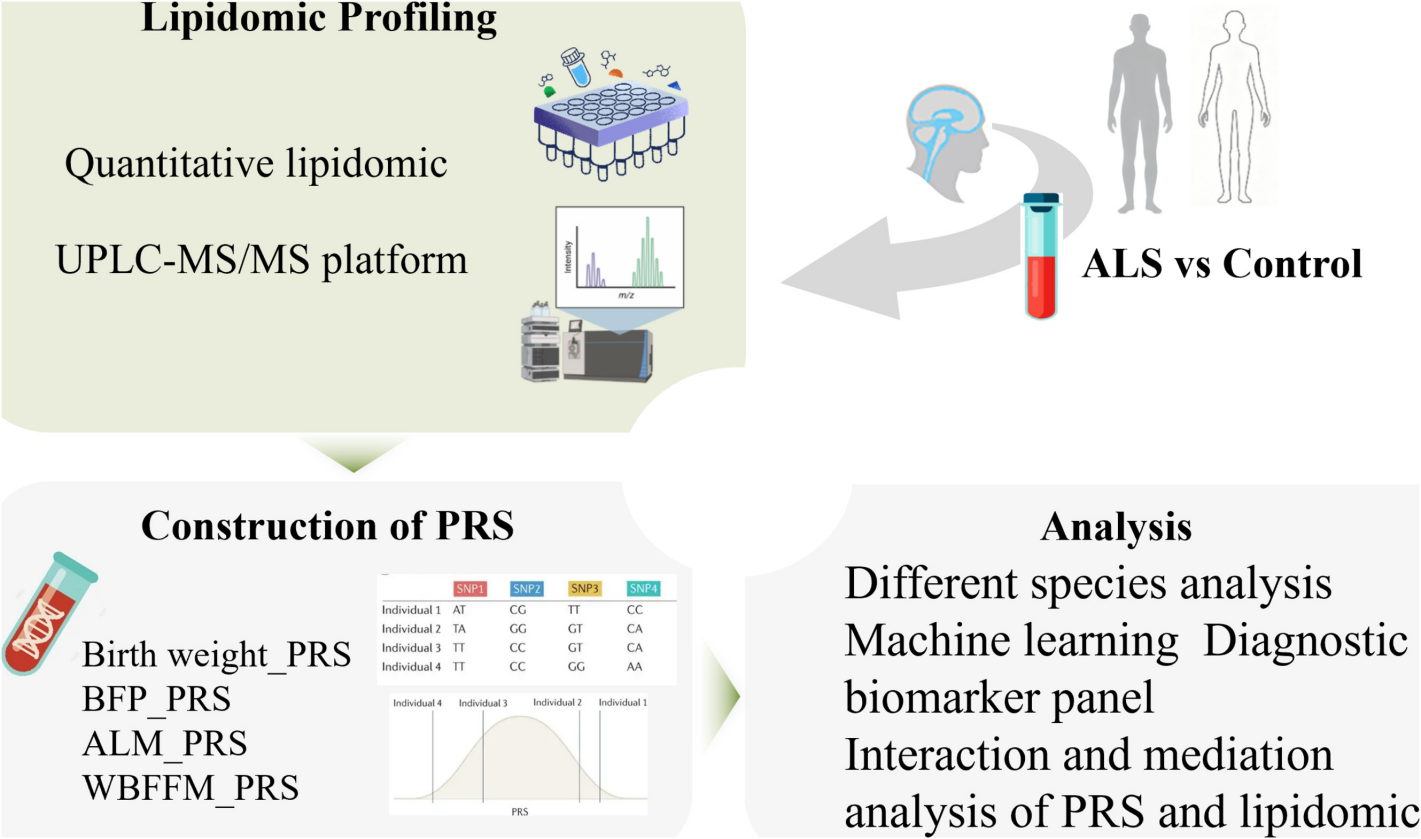
Study design and analytical workflow. Plasma samples from patients with amyotrophic lateral sclerosis (ALS) and neurologically healthy controls underwent quantitative lipidomic profiling using ultraperformance liquid chromatography–tandem mass spectrometry (UPLC‐MS/MS). Polygenic risk scores (PRS) were constructed for four life‐course adiposity‐related traits, including birth weight (BW), body fat percentage (BFP), appendicular lean mass (ALM) and whole‐body fat‐free mass (WBFFM). Downstream analyses included differential lipidomic profiling, machine learning–based candidate biomarker development and PRS–lipid interaction and mediation analyses. ALM, appendicular lean mass; ALS, amyotrophic lateral sclerosis; BFP, body fat percentage; BW, birth weight; PRS, polygenic risk score; UPLC‐MS/MS, ultraperformance liquid chromatography–tandem mass spectrometry; WBFFM, whole‐body fat‐free mass.

### 2.1 Study Participants and Biospecimen Collection

We prospectively enrolled sporadic ALS patients (aged > 18 years) meeting revised El Escorial criteria (clinically definite/probable ALS) within 2 years after symptom onset and sex‐/age‐matched healthy controls from Peking University Third Hospital. Neurologically healthy controls were recruited from the institutional health examination centre during the same study period. Both groups fasted from 10:00 PM before blood collection. Blood was collected at approximately 5:00 AM in patients with ALS (~7‐h fasting) and 8:00 AM in healthy controls (~10‐h fasting). All plasma samples were collected in K_2_EDTA tubes, centrifuged within 2 h (1500 × g, 10 min, 4°C) after blood collection, divided into aliquots and stored at −80°C until lipidomic analysis. Demographic data (sex, age and BMI) and ALS‐specific characteristics—including ALSFRS‐R scores at recruitment, symptom onset (site and timing), diagnostic delay, familial ALS history and current medications—were systematically recorded. The study received ethical approval from Peking University Third Hospital's Institutional Review Board (IRB 00006761), with written informed consent obtained from all participants prior to enrollment. The investigators were blinded to sample allocation during the lipid profiling.

### 2.2 Quantitative Lipidomic Profiling

Quantitative lipidomic profiling was performed by MetWare Biotechnology Co. Ltd. (Wuhan, China) using UPLC‐MS/MS. Samples were shipped on dry ice and stored at −80°C until analysis. The platform consisted of an ExionLC ad UPLC system coupled to a QTRAP 6500 + MS/MS system (SCIEX) [20], with metabolite identification against the proprietary MWLDB v3.0 database covering > 4000 lipid species. Quality control measures included: (1) pooled QC samples injected at regular intervals; (2) internal standards for quantification; and (3) rigorous data preprocessing (see Figure S1), demonstrating excellent technical reproducibility. MS data were acquired in both positive and negative ionization modes with MRM for targeted lipids. A total of 890 lipid species were detected in 182 plasma samples. The LC‐MS data (an 890 × 182 data matrix) were then exported to Microsoft Excel for further analysis.

### 2.3 SNP Genotyping and PRS Construction

Following standard DNA extraction, SNP genotyping was conducted on the MassARRAY MALDI‐TOF System with genotype assignment via TYPER software. Quality control was performed using the CRLMM package in R, excluding SNPs with a call rate < 95%, MAF < 0.01 or deviation from Hardy–Weinberg equilibrium (*p* < 1 × 10^−6^). Subsequently, beta‐weighted PRS for BW (17 SNPs) [21], BFP (12 SNPs) [19], ALM (23 SNPs) [22] and WBFFM (18 SNPs) were calculated in PRSice using published GWAS data at specified *p*‐value thresholds (5 × 10^−8^ or 5 × 10^−8^ or 5 × 10^−5^) [23]. The SNPs and associated genes used are detailed in Table S1.

### 2.4 Differential Metabolite Identification

#### 2.4.1 Analytical Methods

Demographic and clinical characteristics of ALS patients and matched controls across cohorts were compared using Wilcoxon rank‐sum tests (continuous variables) and chi‐square tests (categorical variables).

##### 2.4.1.1 Data Preprocessing and Statistical Analysis

Prior to all downstream analyses, raw lipidomic data were preprocessed to ensure statistical robustness. Missing values were imputed using half of the minimum value for each lipid species. Given its established influence on circulating lipid profiles, BMI was included with age and sex in lipid‐data preprocessing. To address non‐normality and outliers, all lipid abundance data underwent inverse normal transformation (INT), which was performed after regressing out the effects of potential confounders (age, sex and body mass index). This processed dataset was utilized for all subsequent analyses.

Differential abundance analysis between ALS patients and controls was conducted using *t*‐tests on the INT‐transformed residuals. To ensure the identification of both statistically significant and biologically substantial alterations, we applied a dual‐threshold criterion: (1) a false discovery rate (FDR) adjusted *p* value < 0.05 for statistical significance and (2) an absolute Cohen's *d* value > 1.0 as a stringent cut‐off for a large effect size. This approach prioritizes lipid species that demonstrate not only reliable group differences but also considerable magnitude of change, thereby highlighting the most robust metabolic perturbations associated with ALS.

Then within the ALS group, associations of the 890 lipid species with ALSFRS‐R, ΔFS and disease duration (partial correlation analysis) were evaluated to assess the influence of clinical heterogeneity at sampling. Benjamini–Hochberg correction was applied for each clinical characteristic, with FDR < 0.05 considered significant. Exploratory subgroup analyses compared lipid profiles between bulbar versus spinal/limb onset (*t*‐test) and nominal *p* < 0.05 findings reported descriptively after FDR correction for multiple testing.

#### 2.4.2 Construction of Machine Learning–Based ALS Diagnostic Models

Using the significantly differential lipids, we compared logistic regression, random forest and linear and radial basis function support vector machines under stratified fivefold cross‐validation. Random forest–based recursive feature elimination (RFE) was used to derive candidate panels of three to five lipids. To reduce feature‐selection optimism, an additional fivefold internal cross‐validation was performed with three‐feature RFE repeated within each training fold and evaluation restricted to the held‐out fold. Pooled out‐of‐fold predictions were used to calculate the overall ROC AUC, with fold‐specific AUCs summarized as mean ± SD.

#### 2.4.3 Association of Plasma Lipids Profiles With PRS of Life‐Course Adiposity

Linear regression analyses were conducted to assess the associations between life‐course adiposity PRS and plasma lipid levels. The analysis included 175 subjects (102 ALS patients and 73 controls), after excluding 5 samples due to genotyping failure. For each lipid species, the inverse normal transformed residual (adjusted for age, sex and BMI) served as the dependent variable. The independent variables included each of the four adiposity PRS, case–control status (CC) and their interaction term (PRS × CC), with age and sex included as covariates. The interaction term estimated whether the PRS–lipid association differed between ALS patients and controls. Individual interaction effects were considered statistically robust only after FDR correction; uncorrected *p* < 0.05 results were treated as nominal and exploratory.

To assess whether nominal interaction signals were enriched within specific lipid classes, the 78 FDR‐significant ALS‐associated lipids were used as the background set. For each lipid class, over‐representation among the nominal PRS‐by‐disease‐status interaction signals was evaluated using a one‐sided Fisher's exact test, and the resulting *p* values were corrected across lipid classes using the Benjamini–Hochberg method.

Sensitivity analyses of the nominal PRS‐by‐disease‐status interactions were performed with additional case‐specific adjustment for disease duration or ΔFS; these ALS‐specific variables were coded as zero in controls.

#### 2.4.4 Mediation Analysis of Plasma Lipids in the Association Between Life‐Course Adiposity PRS and ALS

To examine whether the association between life‐course adiposity PRS and ALS risk is mediated by alterations in plasma lipid abundance, we conducted a mediation analysis following the causal steps approach proposed by Baron and Kenny [24]. The analysis included 175 subjects and was performed across all 890 quantified lipid features to comprehensively screen for potential mediators. We specified the hypothesized causal pathway as: PRS (X) → Lipid (M) → ALS Case–Control Status (Y). For each lipid, three regression models were fitted: (1) the *total effect* (path c) of PRS on ALS status (logistic regression); (2) the effect of PRS on lipid abundance (path a, linear regression); and (3) the model containing both the *direct effect* (path *c'*) of PRS on ALS status and the effect of the lipid on ALS status (path b, logistic regression). All models were adjusted for age and sex. The significance of the indirect (mediation) effect, calculated as the product of coefficients a × b, was assessed using the Sobel test. A lipid species was considered a significant mediator if the Sobel test *p* value was < 0.05.

All statistical analyses and predictive modelling were performed using R (Version 4.2.1), Python (Version 3.12) and Stata (Version 17.0).

### 2.5 Availability of Data

The data that support the findings of this study are available from the corresponding author, upon reasonable request.

## 3 Results

### 3.1 Descriptive Statistics

The plasmas were collected from 182 subjects (104 ALS patients and 78 neurological‐healthy controls). Baseline clinical characteristics were collected for both cohorts (Table 1). Notably, baseline demographics (age and gender) did not differ significantly between ALS patients and healthy controls. Consequently, variations in lipidomic signatures between groups are more plausibly linked to ALS pathogenesis than to potential confounders including age or sex. Despite the higher BMI in controls compared to ALS subjects (25 vs. 23.4 kg/m^2^; *p* = 0.014), lipid analyses were adjusted for BMI due to its established significant influence on metabolic profiles.

**TABLE 1:** Baseline characteristics of the study population.

Covariate	ALS cases (*n* = 104)	Controls (*n* = 78)	*p*
Age at plasma collection (years)	54.00 (45.75–61.00)	52.00 (48.25–56.75)	0.472
Sex			0.948
Female	41 (39.4%)	32 (41.0%)	
Male	63 (60.6%)	46 (59.0%)	
BMI at the enrollment, kg/m^2^	23.40 (21.88–25.77)	25.00 (22.20–27.48)	0.014
Onset time, months	13.00 (9.00–18.00)	NA	
ALSFRS‐R at plasma collection	42.00 (38.75–45.00)	NA	
El Escorial criteria			
Probable	42 (40.4)	NA	
Definite	62 (59.6)	NA	
Onset segment			
Bulbar	10 (9.6)	NA	
Limbs	94 (90.4)	NA	
Time between symptom onset and diagnosis, months	10.00 (7.75–13.00)	NA	
ΔFS	0.46 (0.22–0.87)	NA	

*Note:* Continuous variables are presented as median (interquartile range [25th–75th percentile]) and categorical variables as *n* (%). The *p* values were calculated using the Wilcoxon rank‐sum test for continuous variables and the chi‐square test for categorical variables; *p* values were not calculated for ALS‐specific variables. ΔFS was calculated as (48 − ALSFRS‐R score at plasma collection)/time from symptom onset to plasma collection (months).

Abbreviations: ΔFS, disease progression rate; ALS, amyotrophic lateral sclerosis; ALSFRS‐R, Amyotrophic Lateral Sclerosis Functional Rating Scale‐Revised; BMI, body mass index; NA, not applicable.

### 3.2 Identification of Differential Metabolites and Lipids

Analytical robustness was confirmed by established quality control metrics (Figure S1). A total of 890 lipid species across 7 major classes were quantified, comprising 40 carnitines, 25 fatty acids, 69 sphingolipids, 401 glycerophospholipids, 327 glycerides, 27 sterol lipids and 1 prenol lipid. The proportion of lipids significantly altered between ALS patients and controls for each class is shown in Figure 2. Volcano plots visualizing the Cohen's *d* (effect size: ALS‐Ctrl) and statistical significance (−log_10_ of FDR‐corrected *p*‐values) for all individual lipid species are presented in Figure 3A–E.

**FIGURE 2:**
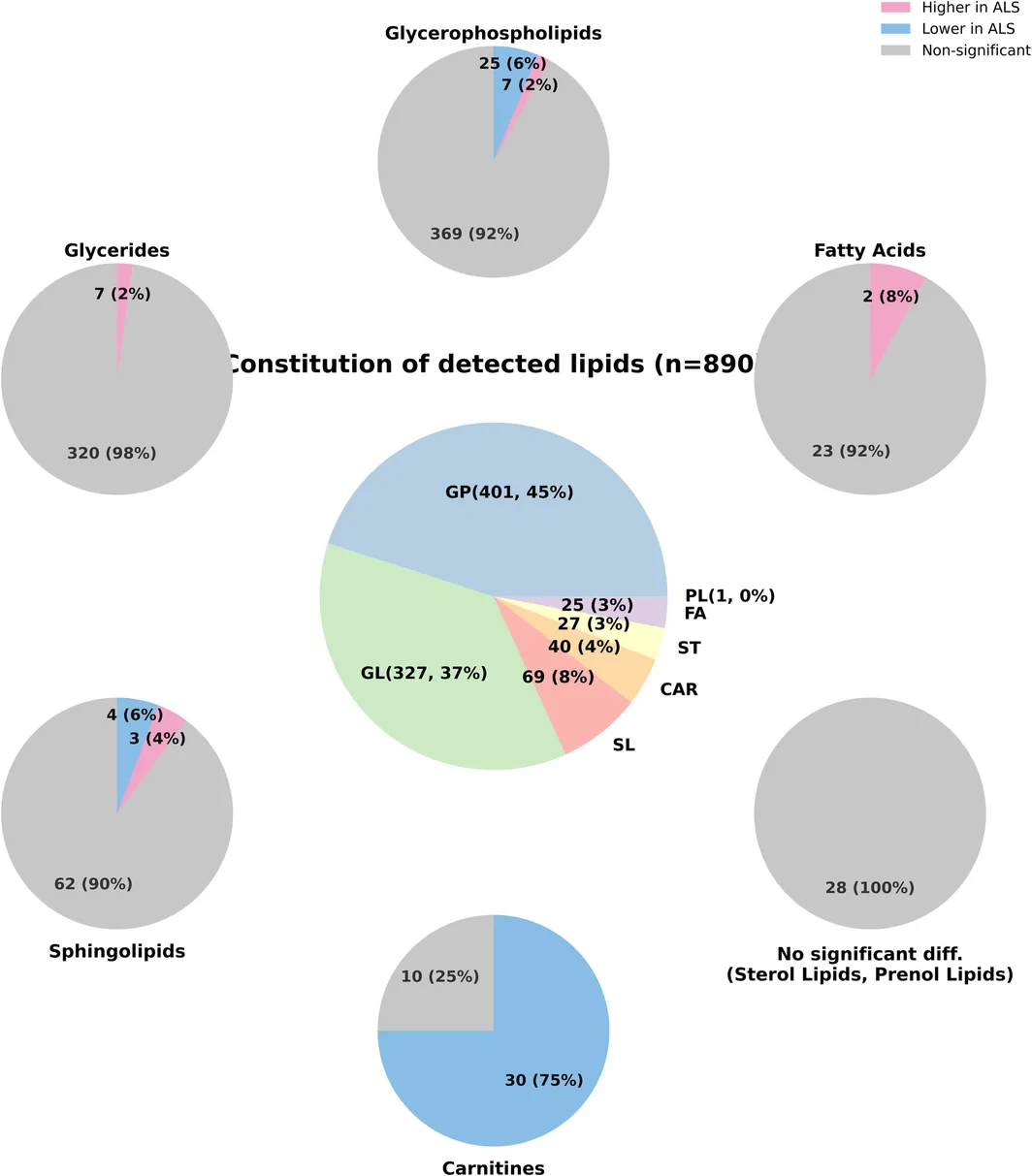
Distribution of lipid classes significantly altered between ALS patients and controls. The central pie chart shows the composition of the 890 detected lipid species across seven major lipid classes. Surrounding pie charts show the numbers and proportions of lipid species that were significantly higher or lower in patients with ALS within each lipid class. Differential lipids were defined by FDR < 0.05 and |Cohen's *d*| > 1.0. Acylcarnitines showed the most prominent reduction, with 30 of 40 detected species (75%) lower in ALS, whereas selected glycerides, fatty acids, glycerophospholipids and sphingolipids showed class‐specific alterations. No significant differences were observed among sterol or prenol lipids. *Note:* Pink indicates lipids higher in ALS, blue indicates lipids lower in ALS, and grey indicates lipids not meeting the significance criteria. Numbers and percentages indicate the number and proportion of lipid species within each class. CAR, carnitines/acylcarnitines; FA, fatty acids; FDR, false discovery rate; GL, glycerides; GP, glycerophospholipids; PL, prenol lipids; SL, sphingolipids; ST, sterol lipids.

**FIGURE 3:**
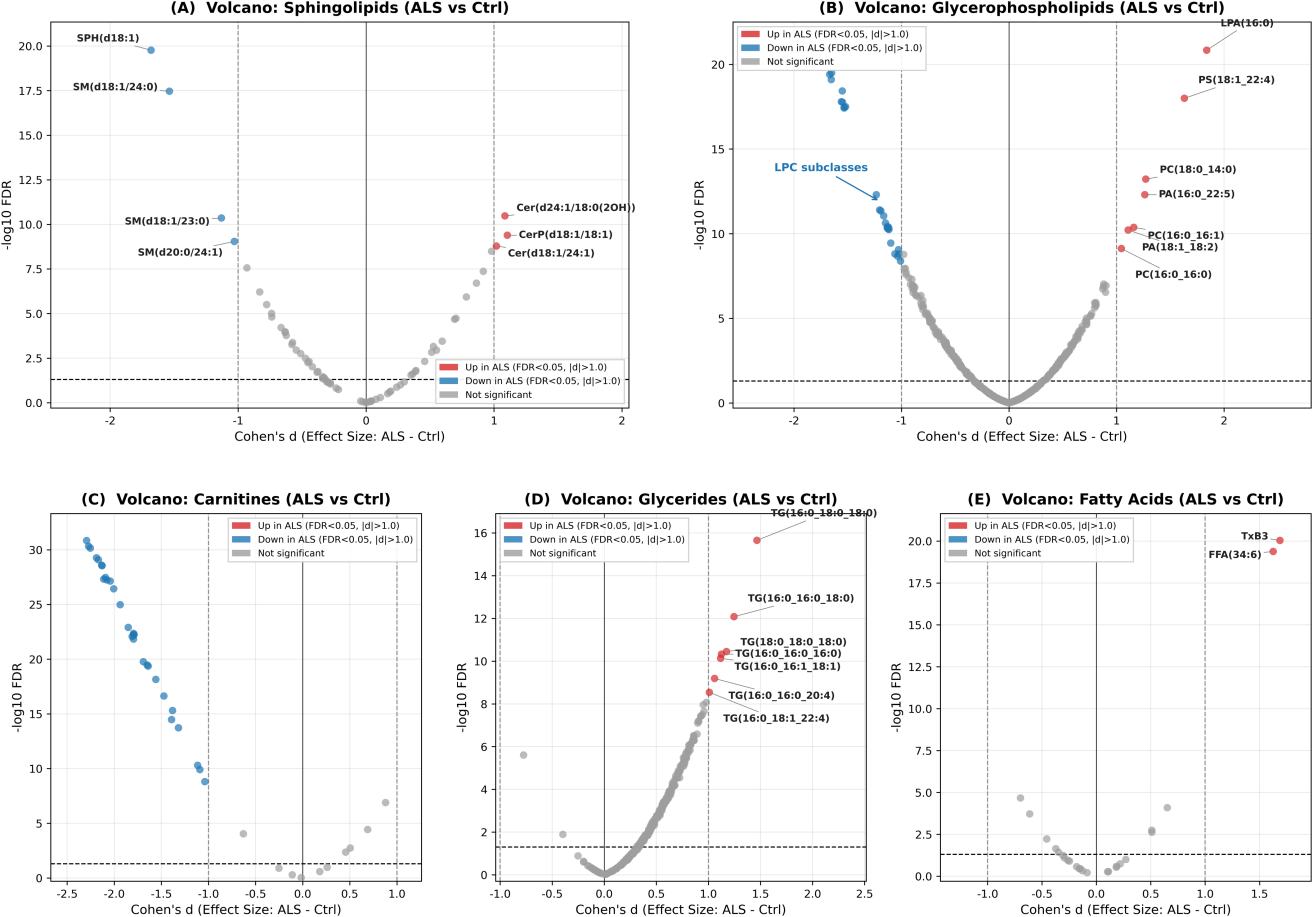
Volcano plots of lipid alterations with ALS compared with controls. Volcano plots show differential lipid abundance for (A) sphingolipids, (B) glycerophospholipids, (C) carnitines, (D) glycerides and (E) fatty acids. The x‐axis represents Cohen's d for the difference between ALS and control groups (ALS − control), and the y‐axis represents −log_10_(FDR). Lipids meeting both FDR < 0.05 and |Cohen's *d*| > 1.0 were considered significantly altered. Positive effect sizes indicate higher levels in ALS, whereas negative values indicate lower levels in ALS. Selected representative lipid species are labelled. *Note:* Red indicates significantly higher lipid levels in ALS, blue indicates significantly lower levels, and grey indicates lipids not meeting both significance criteria. Vertical dashed lines indicate |Cohen's *d*| = 1.0, and the horizontal dashed line indicates FDR = 0.05. ALS, amyotrophic lateral sclerosis; Ctrl, control; FDR, false discovery rate.

Regarding sphingolipids, 4 species (6%) were significantly lower in ALS patients, including SPH(d18:1), SM(d18:1/24:0), SM(d18:1/23:0) and SM(d20:0/24:1). Conversely, 3 species (4%) were higher, including CerP(d18:1/18:1), Cer(d24:1/18:0(2OH)) and Cer(d18:1/24:1).

Among the 25 down‐regulated glycerophospholipids, lysophosphatidylcholines (LPCs) were the most prominently affected, with 22 out of 35 species uniformly depleted. Only one phosphatidylglycerol (PG) and two lysophosphatidylethanolamines (LPEs) were decreased. Seven compounds were significantly up‐regulated: PC(16:0_16:0), PC(16:0_16:1), PC(18:0_14:0), PA(18:1_18:2), PA(16:0_22:5), PS(18:1_22:4) and LPA(16:0).

Notably, medium‐ and long‐chain acylcarnitines exhibited a marked depletion, with 30 out of 40 species (75%) being significantly lower in ALS patients. In contrast, seven specific triglycerides (TGs), accounting for 2% of this subclass, were consistently elevated: TG(16:0_18:0_18:0), TG(16:0_16:0_18:0), TG(18:0_18:0_18:0), TG(16:0_16:0_16:0), TG(16:0_16:1_18:1), TG(16:0_16:0_20:4) and TG(16:0_18:1_22:4). Furthermore, two fatty acids (8%) were up‐regulated: TxB3 and FFA (34:6).

#### 3.2.1 Associations With ALS‐Specific Clinical Characteristics

Using the age‐, sex‐ and BMI‐adjusted lipid residuals, only two associations remained significant after FDR correction in the complete 890‐lipid analysis: Higher ALSFRS‐R scores were associated with lower SM(d18:1/14:0) (β = −0.0727, FDR = 0.0496) and SM(d18:1/18:0) (β = −0.0779, FDR = 0.0496). No FDR‐significant associations were identified for disease duration, ΔFS or site of onset. Within the 78 ALS‐associated lipids, none was significantly associated with ALSFRS‐R, disease duration, ΔFS or site of onset after FDR correction (Table S2).

#### 3.2.2 Machine Learning–Based Diagnostic Model Performance

A heatmap cluster showed that the different lipid levels were distinct between ALS patients and controls, indicating potential value to identify ALS patients from healthy controls (Figure 4A). Using the INT‐transformed data of the 78 significant differential lipids, a parsimonious fixed panel comprising carnitine C12:0, carnitine C10:0 and carnitine C18:2 yielded an AUC of 0.977 under standard stratified fivefold cross‐validation. In the more conservative fold‐specific feature‐selection analysis, RFE was repeated within each training fold and the held‐out fold was used only for evaluation; the pooled out‐of‐fold AUC was 0.958, with a mean fold AUC of 0.959 ± 0.050 (Figure 4B). These estimates represent internal validation only.

**FIGURE 4:**
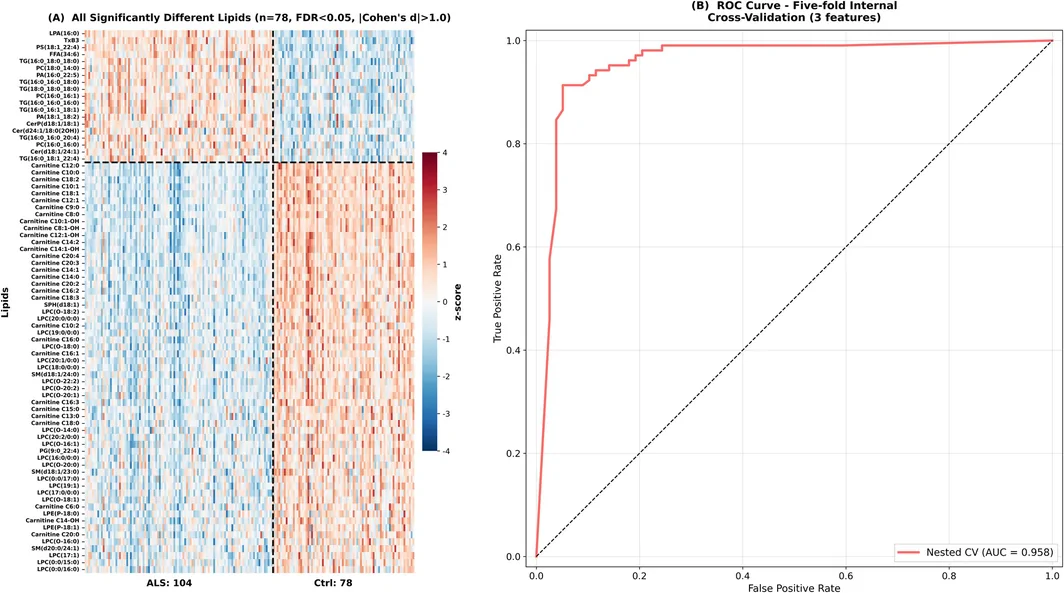
Lipidomic signature and internal evaluation of the candidate diagnostic model. (A) Heatmap of the 78 ALS‐associated lipid species meeting FDR < 0.05 and |Cohen's *d*| > 1.0, illustrating distinct lipidomic patterns between patients with ALS and controls. Lipid abundances are displayed as standardized z‐scores. (B) Receiver operating characteristic (ROC) curve from the additional fivefold internal cross‐validation procedure in which recursive feature elimination selecting three features was repeated separately within each training fold and performance was evaluated exclusively in the corresponding held‐out fold. Pooled out‐of‐fold predictions yielded an overall AUC of 0.958 (mean fold AUC = 0.959 ± 0.050). *Note:* Red and blue in the heatmap indicate relatively higher and lower standardized lipid abundance, respectively. The dashed diagonal line in the ROC plot represents chance‐level discrimination. ALS, amyotrophic lateral sclerosis; AUC, area under the receiver operating characteristic curve; FDR, false discovery rate; RFE, recursive feature elimination; ROC, receiver operating characteristic.

#### 3.2.3 Association of Lipids Profiles With Life‐Course Adiposity PRS

We investigated the associations between four life‐course adiposity PRS and the plasma lipidomic profile. The analysis focused on the 78 lipid species previously identified as differentially abundant between ALS patients and controls. This approach resulted in 312 statistical tests (78 lipids × 4 PRS). After false FDR correction, no direct associations between any PRS and individual lipids remained significant. However, in exploratory analyses examining PRS‐by‐disease status (ALS vs. control) interactions, 16 lipid species showed nominally significant interaction effects. Group‐specific regression plots illustrating the 16 nominal PRS‐by‐disease‐status interaction signals are provided in Figure S2. These comprised 13 acylcarnitines, one sphingomyelin [SM(d18:1/23:0)], one phosphatidylcholine [PC (16:0/16:0)] and one triacylglycerol [TG (16:0/16:1/18:1)] (Table 2). Although no individual PRS‐by‐disease‐status interaction survived FDR correction, acylcarnitines were significantly enriched among the 16 nominal signals (13/16 vs. 30/78 background lipids; Fisher's exact test; OR = 11.47, *p* = 1.30 × 10^−4^, FDR = 6.52 × 10^−4^).

**TABLE 2:** Nominally significantly differential association of the life‐course adiposity risk PRS with lipids between ALS and control.

Feature	PRS	PRS_coef	PRS_p	Interaction_coef	Interaction_p	Adj_R_squared
PC(16:0_16:0)	ALM	0.090	0.050	−0.117	0.049	0.24
Carnitine C14:0	BFP	0.137	0.006	−0.211	0.001	0.45
Carnitine C16:1	BFP	0.109	0.043	−0.198	0.004	0.38
Carnitine C8:0	BFP	0.080	0.087	−0.160	0.008	0.52
Carnitine C10:0	BFP	0.054	0.225	−0.148	0.011	0.56
Carnitine C12:1	BFP	0.059	0.205	−0.146	0.015	0.53
Carnitine C6:0	BFP	0.092	0.118	−0.183	0.017	0.23
Carnitine C12:0	BFP	0.047	0.293	−0.137	0.018	0.56
Carnitine C14‐OH	BFP	0.072	0.229	−0.177	0.023	0.22
Carnitine C14:1	BFP	0.055	0.277	−0.146	0.025	0.44
Carnitine C18:0	BFP	0.105	0.065	−0.159	0.030	0.29
Carnitine C20:4	BW	−0.175	0.027	0.227	0.016	0.48
Carnitine C20:3	BW	−0.137	0.090	0.221	0.022	0.45
SM(d18:1/23:0)	BW	0.205	0.028	−0.229	0.039	0.25
Carnitine C18:3	WBFM	0.141	0.025	−0.217	0.007	0.43
TG(16:0_16:1_18:1)	WBFM	−0.041	0.552	0.175	0.049	0.25

*Note:* Analyses included 175 participants (102 ALS cases and 73 controls) with complete genotyping data. Lipid abundances were residualized for age, sex and BMI and inverse‐normal transformed before analysis. For each lipid–PRS pair, the linear regression model included PRS, disease status, the PRS × disease‐status interaction, age and sex, with controls as the reference group. PRS_coef and PRS_p denote the estimated PRS–lipid association and its nominal *p* value in controls; Interaction_coef and Interaction_p denote the between‐group difference in the PRS–lipid slope (ALS minus controls) and its nominal *p* value. Adj_R_squared is the adjusted *R* ^2^ of the full model. The table includes interactions with nominal *p* < 0.05; none remained significant after false discovery rate correction.

Abbreviations: ALM, appendicular lean mass; ALS, amyotrophic lateral sclerosis; BFP, body fat percentage; BMI, body mass index; BW, birth weight; PC, phosphatidylcholine; PRS, polygenic risk score; SM, sphingomyelin; TG, triglyceride; WBFM, whole‐body fat‐free mass.

The nominal interaction patterns differed across adiposity traits. The BW PRS showed nominal group‐dependent associations with two acylcarnitines (C20:4 and C20:3) and SM(d18:1/23:0). The BFP PRS showed nominal interactions with 10 different acylcarnitines. Nominal interaction was also observed for the ALM PRS with PC(16:0/16:0) and for the WBFFM PRS with acylcarnitine C18:3 and TG(16:0/16:1/18:1). Furthermore, for a subset of six lipids out of the 16, the main effect of the PRS itself (independent of disease status) was nominally significant (*p* < 0.05; Table 2).

Sensitivity analyses showed limited changes in the nominal PRS‐by‐disease‐status interaction pattern. After separate adjustment for disease duration and ΔFS, 14 and 15 of the 16 original nominal interactions, respectively, remained at *p* < 0.05, including all 13 acylcarnitine signals; none survived multiple‐testing correction (Table S3).

Moreover, we expanded the analysis to include all 890 detected lipid species, conducting 890 × 4 pair‐wise association tests. After FDR correction, no significant direct associations were observed. However, a nominally significant interaction (PRS × disease status, *p* < 0.05) was identified for 185 lipid species. The distribution of these lipids across major subclasses associated with each PRS is visualized in a radar chart (Figure 5). Specifically, lipids interacting with BW PRS were predominantly glycerophospholipids (62%) and sphingolipids (23%), with smaller proportions of carnitines (9%) and glycerides (6%). For BFP PRS, the interacting lipids were primarily glycerides (38%) and glycerophospholipids (38%), followed by carnitines (18%) and fatty acids (6%). In contrast, lipids associated with ALM PRS were overwhelmingly glycerophospholipids (73%). Finally, interactions for WBFFM PRS were mainly composed of glycerides (62%), with secondary contributions from glycerophospholipids (18%), sterol lipids (7%), carnitines (5%), sphingolipids (4%) and fatty acids (4%).

**FIGURE 5:**
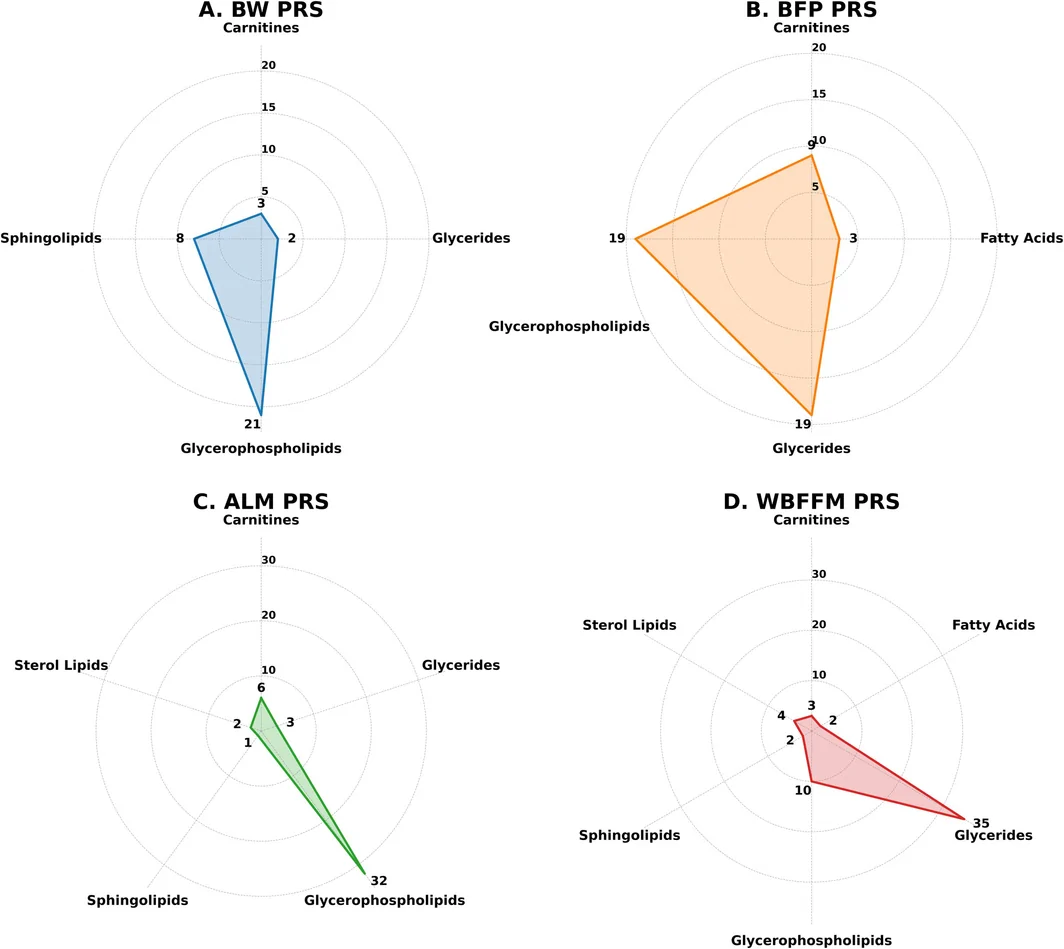
Lipid‐class distribution of nominal PRS‐by‐disease‐status interaction signals across the complete lipidome. Radar plots show the lipid‐class composition of nominal PRS‐by‐disease‐status interaction signals (*p* < 0.05) identified across all 890 lipid species for PRS of (A) birth weight, (B) body fat percentage, (C) appendicular lean mass and (D) whole‐body fat‐free mass. The radial values indicate the number of nominal interaction signals within each lipid class. The distribution of nominal signals differed across adiposity‐related PRS; however, none of the individual interactions survived FDR correction. *Note:* These analyses are exploratory, and the displayed lipid‐class distributions should not be interpreted as statistically confirmed pathway enrichment. ALM, appendicular lean mass; BFP, body fat percentage; BW, birth weight; FDR, false discovery rate; PRS, polygenic risk score; WBFFM, whole‐body fat‐free mass.

### 3.3 Mediation Results

Our mediation analysis, designed to systematically assess whether any of the 890 quantified blood lipids mediated the relationship between the PRS and ALS risk, yielded null findings. We employed Sobel tests to screen the full spectrum of lipids for potential mediating effects. The analysis was constrained by the fact that none of the four life‐course adiposity PRS demonstrated a statistically significant total effect on ALS risk in the initial association models.

## 4 Discussion

This quantitative lipidomic study delineates a distinct profile of circulating lipid dysregulation in ALS, with 78 FDR‐significant, large‐effect alterations characterized by prominent acylcarnitine depletion and coordinated changes in sphingolipids, glycerophospholipids and triglycerides. A three‐acylcarnitine panel showed high discriminatory performance in internal cross‐validation, supporting the biomarker potential of these metabolic alterations. Exploratory PRS‐by‐disease‐status analyses and class‐level enrichment of acylcarnitines further suggest a possible relationship between adiposity‐related genetic background and carnitine‐associated metabolic variation in ALS. Together, these findings highlight systemic lipid metabolic dysregulation in ALS and provide complementary biomarker and genetic perspectives.

Previously metabolomic and multi‐omics investigations in ALS have collectively revealed multifaceted lipid dysregulation, with recurrent alterations involving fatty acids, acylcarnitines, sphingolipids, glycerophospholipids and glycerolipids across independent cohorts and biological compartments [15, 25, 26, 27, 28]. These findings are broadly consistent with our results at the pathway level, particularly the marked depletion of circulating acylcarnitines and LPCs, selective remodelling of sphingomyelins and ceramides and elevation of several triglyceride species. Nevertheless, the direction and magnitude of individual lipid changes have varied across studies, likely reflecting differences in analytical platforms, fasting and nutritional status, disease stage, genetic background, cohort composition and biological compartment. Accordingly, circulating lipid concentrations, particularly acylcarnitines, should be interpreted as steady‐state markers of altered systemic lipid handling rather than direct measures of intracellular metabolic flux. Previous studies have also reported alterations in circulating lipoprotein composition in ALS, including higher LDL‐1 and lower IDL‐B subfractions, although associations of conventional lipids and apolipoproteins with disease severity and survival have been heterogeneous across cohorts [16, 29]. Accordingly, our lipidomic findings cannot distinguish changes in lipoprotein particle abundance or distribution from remodelling of the lipid cargo carried by these particles.

Comparison with published CSF and CNS‐tissue lipidomic studies showed concordance mainly at the lipid‐class and pathway levels rather than at the level of individual species. CSF studies have implicated phosphatidylcholines, sphingomyelins, ceramides, glucosylceramides and triglycerides in ALS and disease progression [30, 31], whereas paired plasma–CSF analyses showed minimal overlap in individual lipid species and even opposite patterns for several triglycerides [31]. Postmortem CNS studies likewise reported alterations in sphingolipids, phospholipids, triglycerides and cholesteryl esters, although the direction of change varied across tissues [32, 33]. Multi‐omic analysis of vulnerable motor neurons further implicated intrinsic lipid metabolic abnormalities, including arachidonic acid and 5‐lipoxygenase pathways [25]. Collectively, these findings support shared disruption of lipid metabolism across peripheral and CNS compartments while emphasizing substantial compartment specificity. Notably, the broad plasma acylcarnitine depletion observed in our cohort has not been consistently reported in CSF or CNS tissue and may therefore predominantly reflect systemic rather than CNS‐specific metabolic alterations.

Among these alterations, sphingolipid dysregulation has emerged as a recurrent feature of ALS [25, 26, 27, 28]. The sphingomyelin–ceramide axis is closely involved in membrane homeostasis, inflammatory signalling and cellular stress. Genetic evidence further supports the relevance of this pathway: Pathogenic SPTLC1 variants causing juvenile ALS lead to dysregulated de novo sphingolipid synthesis [34, 35]. In our sporadic ALS cohort, several ceramides were increased, whereas the detected sphingosine and several sphingomyelins were reduced. This pattern differs from the excessive sphingolipid synthesis observed in SPTLC1‐associated juvenile ALS and may reflect distinct mechanisms of sphingolipid remodelling in sporadic disease. Increased conversion of sphingomyelin to ceramide under inflammatory or oxidative stress represents one possible explanation, although plasma measurements alone cannot determine the responsible enzymatic pathway or tissue source.

The 30 of 40 detected species (75%) acylcarnitines in this study were uniformly depleted in ALS patients. This finding is supported by accumulating evidence linking carnitine‐related metabolism to ALS. A recent MR study tested the causal effects of about one thousand plasma metabolites on ALS, and carnitine derivatives [36] were observed protective effects for ALS [37]. Recently, a longitudinal metabolomics evidence indicates that carnitine metabolism that correlated with disease severity and progression [15]. They also provided evidence from MR design that these impaired carnitine and short‐chain acylcarnitine metabolism may be genetically linked to ALS, along with various antioxidant derivatives [15]. Another earlier cross‐sectional study showed the lower serum level of L‐carnitine, the higher severity of ALS [38]. Medium‐ and long‐chain acylcarnitines participate in mitochondrial fatty acid transport and therefore provide an indirect window into systemic fatty acid handling. However, their circulating concentrations are determined by multiple processes, including synthesis, tissue uptake, mitochondrial utilization, transport and clearance. Thus, the observed depletion cannot by itself establish impaired β‐oxidation or CNS‐specific mitochondrial dysfunction. Although an initial RCT suggested acetylcarnitine supplementation may improve self‐sufficiency in ALS patients versus placebo [39], its full therapeutic potential awaits confirmation from a larger, ongoing Phase II/III trial designed to assess both biological and clinical outcomes [40].

The concomitant elevation of selected triglycerides may nevertheless be compatible with broader remodelling of systemic lipid utilization and storage in ALS. Beyond mitochondrial fatty acid handling, a complementary neuroendocrine mechanism has recently been proposed by Krishnamurthy et al., linking mutation‐specific lipid abnormalities and altered systemic ANGPTL‐3/4 signalling to hypothalamic dysfunction and neuroinflammation [41]. Through regulation of lipoprotein lipase, the ANGPTL system may influence triglyceride hydrolysis and lipid partitioning across peripheral tissues. Notably, the reported ANGPTL and triglyceride phenotypes varied across ALS genetic subtypes, further highlighting the heterogeneity of systemic lipid remodelling in ALS. As ANGPTL proteins and lipoprotein‐lipase activity were not assessed in our cohort, the relevance of this pathway to our findings remains hypothesis‐generating. Collectively, these findings highlight lipid metabolism as an important component of ALS pathophysiology and support further investigation of its potential diagnostic and therapeutic relevance.

Using machine learning analysis, we identified a parsimonious candidate panel centred on three acylcarnitines (C12:0, C10:0 and C18:2). When feature selection was repeated within each training fold, the three‐feature procedure retained high internal discrimination (overall out‐of‐fold AUC = 0.958; mean fold AUC = 0.959 ± 0.050), although the estimate was lower than the original standard cross‐validated AUC of 0.977. This attenuation is consistent with reduced feature‐selection optimism. However, the analysis remains internal to a single‐centre cohort, and the initial 78 candidate lipids were identified using the full dataset; therefore, residual optimism and cohort‐specific effects cannot be excluded. Accordingly, the model should be regarded as an exploratory candidate biomarker rather than a clinically ready diagnostic tool. Independent multicentre validation using harmonized quantitative assays and clinically relevant ALS‐mimic controls is required to establish reproducibility, disease specificity, calibration and clinical utility.

Regarding the interaction analysis, although no individual interaction survived correction for multiple testing, the significant class‐level over‐representation of acylcarnitines suggests that the nominal signals were not randomly distributed across lipid classes. This pattern provides hypothesis‐generating support for a potential interplay between adiposity‐related genetic background and carnitine‐associated metabolic phenotypes in ALS. The finding that adiposity PRS, especially those for BFP and BW, differentially modulate carnitine levels in patients versus controls suggests that an individual's genetic background for fat storage may influence the severity or presentation of the underlying bioenergetic deficit in ALS. Although evidence linking BW or other fetal growth parameters to ALS remains sparse and inconsistent, with a possible non‐linear association reported in one prospective study but no clear evidence from our previous Mendelian randomization study or a recent case–control study [42, 43, 44]. Furthermore, when the interaction analysis was extended to all 890 detected lipid species, nominal PRS‐by‐disease‐status signals showed differing lipid‐class distributions across adiposity traits; however, none survived multiple‐testing correction. The nominal pattern of ALM PRS with glycerophospholipids may be compatible with variation in membrane‐related lipid metabolism, whereas the nominal signals involving WBFFM PRS and BFP PRS with glycerides may reflect differences in pathways related to energy storage and mobilization. Nominal interactions involving lipids previously implicated in ALS, including SM(d18:1/23:0) with BW PRS and PC(16:0_16:0) with ALM PRS, further raise the possibility that adiposity‐related genetic background may be associated with variation in selected lipid metabolic phenotypes. Collectively, these observations should be regarded as exploratory rather than evidence of established biological modulation by adiposity PRS. A previous pilot study using correlation analysis reported associations between certain metabolomic profiles and BMI trajectories [1]. Despite this, our observed patterns, although not surviving multiple testing correction, remain of interest as hypothesis‐generating findings that merit further discussion.

No significant mediation effects were identified. This null result can likely be attributed to the limited sample size available for this specific analysis, as mediation studies typically require larger cohorts to achieve sufficient statistical power.

The principal strength of this study lies in the comprehensive and quantitative characterization of the plasma lipidome in a well‐defined cohort of patients with definite or probable ALS. Using stringent statistical criteria combining FDR correction with a large effect‐size threshold, we identified 78 robust ALS‐associated lipid alterations spanning acylcarnitines, sphingolipids, glycerophospholipids and triglycerides, thereby providing a coherent view of systemic lipid metabolic disturbances in ALS. A further strength is the integration of life‐course adiposity‐related polygenic risk scores with lipidomic profiling, which allowed us to explore whether inherited predisposition to body composition may modify lipid metabolic patterns in the context of ALS. Although individual PRS‐by‐disease‐status interactions were nominal, the significant enrichment of acylcarnitines among these signals provided convergent, hypothesis‐generating evidence implicating carnitine‐related metabolism. In addition, we systematically evaluated the potential influence of clinical heterogeneity by examining ALSFRS‐R, disease duration, progression rate and site of onset and by performing subgroup and sensitivity analyses. Although selected lipid species have previously been associated with ALS phenotype and progression, such associations have varied across studies; in our cohort, the absence of FDR‐significant associations among the 78 principal ALS‐associated lipids suggests that the core case–control lipidomic signature was not primarily driven by ALSFRS‐R, disease duration, ΔFS or site of onset at sampling. The relative stability of the principal lipidomic and PRS‐related patterns across these analyses supports the robustness of the main cross‐sectional findings and helps distinguish the core ALS‐associated lipid signature from variation attributable to measured clinical characteristics.

Several limitations should be acknowledged. First, the diagnostic classifier was evaluated only by internal cross‐validation, which assessed internal reproducibility rather than external generalizability. External validation in independent multicentre cohorts, including ALS‐mimicking disorders, is therefore required. Second, the relatively modest sample size limited statistical power for mediation and interaction analyses. Consistent with this limitation, none of the individual PRS‐by‐disease‐status interactions survived FDR correction, and the observed class‐level enrichment of acylcarnitines should be regarded as exploratory and hypothesis‐generating. Third, our findings represent a systemic plasma lipidomic signature consistent with altered fatty acid transport or utilization, but the responsible tissue compartments cannot be determined from circulating measurements alone. Plasma lipidomics may therefore not directly reflect CNS metabolism; although CSF data were available for a subset of patients (*n* = 39), the lack of appropriate neurological controls precluded robust cross‐compartmental analyses. Fourth, residual pre‐analytical and metabolic confounding cannot be excluded. ALS patients and controls differed in sampling time and fasting duration, whereas metabolic comorbidities, dyslipidaemia, obesity and lipid‐lowering medication use were not prespecified exclusion criteria. Conventional lipid panels, apolipoproteins and quantitative lipoprotein particle profiles were also unavailable, limiting attribution of the observed lipidomic changes to specific lipoprotein pathways.

## Funding

This work was supported by the National Natural Science Foundation of China (grant 82101490 to L.Z.; grants 81873784 and 82071426 to D.F.), the Clinical Cohort Construction Program of Peking University Third Hospital (BYSYDL2019002), the National Clinical Key Specialty Construction Program of China (2024), the Peking University Clinical Scientist Training Program and the Young Elite Scientists Sponsorship Program of the Beijing High Innovation Plan (20250824 to L.Z.).

## Conflicts of Interest

The authors declare no conflicts of interest.

## Supporting information


**Figure S1:** Quality control and data preprocessing for plasma lipid profiling. Initially, 895 known lipids were identified across the four groups comprising 182 samples. Missing values were imputed using half of the minimum value for each lipid species. Subsequently, the coefficient of variation (CV) was calculated for quality control (QC) samples, and only metabolites and lipids with a CV < 0.3 were retained for downstream analyses. Nearly 100% of lipids met this QC criterion (CV < 0.3), indicating high data stability and reliability (A). A total of 890 lipids were used for differential abundance and subsequent analyses. High QC sample correlation coefficients (close to 1) further confirmed data stability and quality (B). The total ion chromatograms (TICs) of all QC samples were overlaid to assess retention time consistency and signal intensity stability across analytical batches. The TIC overlay plots revealed minimal variability in both peak retention times and intensity profiles, demonstrating robust instrument performance and high reproducibility of the metabolomic and lipidomic data acquisition processes (C,D). During the study's analysis, data scaling was performed using unit variance scaling (UV) and mean centring.
**Figure S2:** Group‐specific associations underlying the nominal PRS‐by‐disease‐status interaction signals. Scatterplots show the associations between life‐course adiposity polygenic risk scores (PRS) and plasma lipid levels for the 16 lipids with nominally significant PRS‐by‐disease‐status interactions. Red and blue points represent patients with ALS and healthy controls, respectively, with dashed lines indicating group‐specific linear fits. Lipid values are age‐, sex‐ and BMI‐adjusted inverse‐normal‐transformed residuals. The nominal interaction *p* value and corresponding FDR‐adjusted *p* value are shown above each panel. None of the individual interaction effects remained significant after FDR correction. ALM, appendicular lean mass; ALS, amyotrophic lateral sclerosis; BFP, body fat percentage; BW, birth weight; PRS, polygenic risk score; WBFFM, whole‐body fat‐free mass.
**Table S1:** SNPs and associated genes used for construction of the life‐course adiposity polygenic risk scores.
**Table S2:** Within‐ALS associations with clinical characteristics and exploratory subgroup results.
**Table S3:** Sensitivity analyses of the 16 nominal PRS‐by‐disease‐status interactions under alternative clinical covariate adjustments.

## Data Availability

The data that support the findings of this study are available on request from the corresponding author. The data are not publicly available due to privacy or ethical restrictions.
