# Supplementary material for: Plasma Lipidome Is Dysregulated in Amyotrophic Lateral Sclerosis and Associated With Life‐Course Adiposity Polygenic Risk
Source: J Cachexia Sarcopenia Muscle. 2026 Oct 21;17(5):e70410. doi: 10.1002/jcsm.70410 (PMC13685669; doi:10.1002/jcsm.70410)
Supplement: Supplementary file 1 [file JCSM-17-e70410-s001.docx]

**Supplemental Figures and Tables**

**Supplementary Fig. 1: Quality control and data preprocessing for plasma lipid profiling.** Initially, 895 known lipids were identified across the four groups comprising 182 samples. Missing values were imputed using half of the minimum value for each lipid species. Subsequently, the coefficient of variation (CV) was calculated for quality control (QC) samples, and only metabolites and lipids with a CV < 0.3 were retained for downstream analyses. Nearly 100% of lipids met this QC criterion (CV < 0.3), indicating high data stability and reliability (**A**). A total of 890 lipids were used for differential abundance and subsequent analyses. High QC sample correlation coefficients (close to 1) further confirmed data stability and quality (**B**). The total ion chromatograms (TICs) of all QC samples were overlaid to assess retention time consistency and signal intensity stability across analytical batches. The TIC overlay plots revealed minimal variability in both peak retention times and intensity profiles, demonstrating robust instrument performance and high reproducibility of the metabolomic and lipidomic data acquisition processes (**C, D**). During the study's analysis, data scaling was performed using unit variance scaling (UV) and mean centering.

A CV-ECDF. B QC_correlation.

C TIC-N.

D TIC-P.

**Supplementary Fig. 2. Group-specific associations underlying the nominal PRS-by-disease-status interaction signals.**
Scatterplots show the associations between life-course adiposity polygenic risk scores (PRS) and plasma lipid levels for the 16 lipids with nominally significant PRS-by-disease-status interactions. Red and blue points represent patients with ALS and healthy controls, respectively, with dashed lines indicating group-specific linear fits. Lipid values are age-, sex-, and BMI-adjusted inverse-normal-transformed residuals. The nominal interaction *P* value and corresponding FDR-adjusted *P* value are shown above each panel. None of the individual interaction effects remained significant after FDR correction. BFP, body fat percentage; BW, birth weight; ALM, appendicular lean mass; WBFFM, whole-body fat-free mass; PRS, polygenic risk score; ALS, amyotrophic lateral sclerosis.

**Supplementary Table 1.** **SNPs and associated genes used for construction of the life-course adiposity polygenic risk scores.**

| Traits | Rsid | Effect_allele | Other_allele | bata | se | P | Nearest gene |
| --- | --- | --- | --- | --- | --- | --- | --- |
| Birth weight |  |  |  |  |  |  |  |
|  | rs10181515 | T | C | 0.021 | 0.005 | 1.30E-05 | LOC646736/COL4A4/IRS1 |
|  | rs10872678 | T | C | 0.028 | 0.005 | 8.20E-10 | ESR1 |
|  | rs11042596 | T | G | 0.027 | 0.004 | 1.60E-09 | INS-IGF2 |
|  | rs11055030 | G | C | 0.022 | 0.005 | 1.00E-06 | APOLD1 |
|  | rs116807401 | C | T | 0.088 | 0.016 | 7.00E-08 | PABPC4L |
|  | rs13266210 | A | G | 0.03 | 0.005 | 3.10E-09 | ANK1 |
|  | rs138715366 | C | T | 0.235 | 0.022 | 1.40E-25 | YKT6/GCK |
|  | rs1547669 | G | A | 0.018 | 0.004 | 9.30E-06 | MLN |
|  | rs222857 | T | C | 0.026 | 0.004 | 5.80E-10 | CLDN7/SLC2A4 |
|  | rs2551347 | T | C | 0.029 | 0.005 | 2.20E-09 | KLHL29 |
|  | rs40434 | G | A | 0.017 | 0.004 | 4.80E-05 | SLC6A2 |
|  | rs41355649 | G | A | 0.042 | 0.008 | 4.50E-07 | CEBPA |
|  | rs4144829 | C | T | 0.032 | 0.005 | 1.10E-11 | LCORL/DCAF16 |
|  | rs7076938 | T | C | 0.029 | 0.005 | 2.90E-10 | ADRB1 |
|  | rs7819593 | C | T | 0.023 | 0.005 | 2.10E-06 | ZFPM2 |
|  | rs7968682 | G | T | 0.037 | 0.004 | 4.90E-20 | HMGA2 |
|  | rs9909342 | A | G | 0.019 | 0.004 | 6.70E-06 | WSB1 |
| BFP |  | | | | | | |
|  | rs1558902 | A | T | 0.051 | 0.005 | 3.8×10-27 | FTO |
|  | rs2943652 | C | T | 0.034 | 0.005 | 1.5×10-12 | IRS1 |
|  | rs6567160 | C | T | 0.034 | 0.005 | 1.3×10-10 | MC4R |
|  | rs6755502 | C | T | 0.039 | 0.006 | 1.4×10-10 | TMEM18 |
|  | rs6738627 | A | G | 0.03 | 0.005 | 5.7×10-9 | COBLL1-GRB14 |
|  | rs693839 | C | T | 0.028 | 0.005 | 6.6×10-9 | SPRY2 |
|  | rs6857 | C | T | 0.048 | 0.008 | 6.6×10-9 | TOMM40-APOE |
|  | rs4788099 | G | A | 0.027 | 0.005 | 1.2×10-8 | TUFM-SH2B1 |
|  | rs9906944 | C | T | 0.033 | 0.006 | 2.9×10-8 | IGF2BP1 |
|  | rs543874 | G | A | 0.032 | 0.006 | 4.5×10-8 | SEC16B |
|  | rs3761445 | G | A | 0.024 | 0.005 | 1.7×10-7 | PICK1-PLA2G6 |
|  | rs757318 | C | A | 0.024 | 0.005 | 2.1×10-7 | CRTC1 |
| ALM |  | | | | | | |
|  | rs143384 | G | A | 0.073 | 0.002 | 1.00E-200 | GDF5 |
|  | rs6860245 | C | G | 0.059 | 0.002 | 9.66E-160 | SLC12A2-DT |
|  | rs3116602 | G | T | -0.061 | 0.002 | 9.53E-155 | DLEU1 |
|  | rs2871960 | C | A | 0.047 | 0.002 | 2.17E-135 | ZBTB38 |
|  | rs1472852 | A | C | -0.064 | 0.003 | 8.22E-135 | LCORL |
|  | rs9388490 | T | C | 0.046 | 0.002 | 1.33E-130 | CENPW,MIR588 |
|  | rs72656010 | C | T | -0.067 | 0.003 | 7.31E-126 | PLAG1 |
|  | rs76895963 | G | T | 0.164 | 0.007 | 8.22E-112 | CCND2-AS1 |
|  | rs42039 | T | C | 0.048 | 0.002 | 3.53E-106 | CDK6 |
|  | rs66922415 | G | A | 0.049 | 0.002 | 1.10E-104 | RNU4-17P |
|  | rs2236406 | C | T | 0.039 | 0.002 | 1.26E-87 | PTCH1 |
|  | rs10483727 | C | T | -0.037 | 0.002 | 6.73E-80 | SALRNA1 |
|  | rs905938 | C | T | 0.039 | 0.002 | 8.43E-77 | DCST2 |
|  | rs7689420 | C | T | 0.047 | 0.003 | 1.50E-76 | HHIP |
|  | rs11107115 | G | A | 0.041 | 0.002 | 5.36E-69 | na |
|  | rs12713004 | G | A | 0.037 | 0.002 | 2.40E-68 | KLHL29 |
|  | rs1260326 | C | T | 0.032 | 0.002 | 6.16E-64 | GCKR |
|  | rs2871865 | G | C | -0.049 | 0.003 | 3.40E-62 | IGF1R |
|  | rs2885697 | T | G | -0.032 | 0.002 | 9.21E-60 | SCMH1 |
|  | rs2194411 | A | G | 0.044 | 0.003 | 2.43E-54 | TRA2B |
|  | rs11042717 | C | T | -0.029 | 0.002 | 4.04E-53 | SBF2 |
|  | rs72801843 | A | T | 0.031 | 0.002 | 8.83E-52 | RBL2 |
|  | rs72832859 | G | C | 0.036 | 0.002 | 2.36E-50 | PLCD3 |
| WBFFM |  | | | | | | |
|  | rs56094641 | G | A | 0.036 | 0.001 | 1.00E-178 | FTO |
|  | rs143384 | G | A | 0.035 | 0.001 | 1.50E-171 | GDF5 |
|  | rs9853018 | T | C | 0.030 | 0.001 | 6.30E-128 | ZBTB38 |
|  | rs2744956 | C | T | 0.038 | 0.002 | 2.90E-99 | ILRUN |
|  | rs7740107 | A | T | -0.029 | 0.001 | 1.10E-95 | L3MBTL3 |
|  | rs6743060 | A | C | 0.034 | 0.002 | 1.10E-93 | TMEM18 |
|  | rs72656010 | C | T | -0.037 | 0.002 | 2.80E-88 | PLAG1 |
|  | rs76895963 | G | T | 0.095 | 0.005 | 2.80E-87 | CCND2-AS1 |
|  | rs10269774 | A | G | 0.026 | 0.001 | 4.70E-87 | CDK6 |
|  | rs2225226 | T | C | -0.030 | 0.002 | 3.10E-86 | na |
|  | rs34517439 | A | C | 0.037 | 0.002 | 3.30E-84 | GIPC2 |
|  | rs4240326 | G | A | -0.021 | 0.001 | 3.70E-65 | ANAPC10 |
|  | rs7235010 | A | G | 0.026 | 0.002 | 5.30E-65 | CABLES1 |
|  | rs8077653 | G | T | 0.022 | 0.001 | 1.40E-63 | CD79B |
|  | rs3753639 | C | T | 0.023 | 0.001 | 5.80E-56 | ZBTB7B |
|  | rs7033487 | C | T | -0.023 | 0.002 | 4.50E-51 | PAPPA |
|  | rs1582931 | A | G | -0.019 | 0.001 | 8.70E-51 | CEP120 |
|  | rs7952436 | T | C | -0.034 | 0.002 | 6.70E-50 | KDM2A |

Notes: Effect allele refers to the allele corresponding to the reported effect estimate (β) in the source GWAS. β, effect size estimate; SE, standard error; P, association P value; BFP, body fat percentage; ALM, appendicular lean mass; WBFFM, whole-body fat-free mass; SNP, single-nucleotide polymorphism; GWAS, genome-wide association study; NA, not available.

**Supplementary Table 2. Within-ALS associations with clinical characteristics and exploratory subgroup results.**

A. FDR-significant associations in the complete lipidome (890 lipid species; n = 2 associations)

| Clinical variable | Lipid species | Coefficient | Nominal P value | FDR | Among 78 ALS-associated lipids |
| --- | --- | --- | --- | --- | --- |
| ALSFRS-R | SM(d18:1/14:0) | -0.0727 | 1.05E-4 | 0.0496 | No |
| ALSFRS-R | SM(d18:1/18:0) | -0.0779 | 1.11E-4 | 0.0496 | No |

B. Nominal associations in the bulbar-versus-spinal/limb onset comparison (P < 0.05; n = 19)

| Lipid species | t statistic | Nominal P value | FDR |
| --- | --- | --- | --- |
| Cholesterol | 4.066 | 0.0016 | 0.8970 |
| PC(O-16:0_14:0) | 3.358 | 0.0035 | 0.8970 |
| PE(18:2_22:5) | 3.070 | 0.0050 | 0.8970 |
| Carnitine C8:1-OH | 2.794 | 0.0124 | 0.8970 |
| HexCer(t14:0/24:2(2OH)) | 2.845 | 0.0151 | 0.8970 |
| Carnitine C10:1 | 2.699 | 0.0184 | 0.8970 |
| Carnitine C9:0 | 2.704 | 0.0204 | 0.8970 |
| TG(24:1_18:2_18:2) | 2.574 | 0.0230 | 0.8970 |
| PA(18:0_22:6) | 2.563 | 0.0236 | 0.8970 |
| PC(22:6_17:0) | 2.602 | 0.0249 | 0.8970 |
| SM(d18:0/16:0) | 2.581 | 0.0249 | 0.8970 |
| Carnitine C12:1-OH | 2.332 | 0.0326 | 0.8970 |
| PE(O-18:1_22:5) | -2.338 | 0.0403 | 0.8970 |
| PI(18:1_17:2) | -2.173 | 0.0426 | 0.8970 |
| TG(18:1_18:2_22:0) | 2.212 | 0.0439 | 0.8970 |
| TG(16:1_18:1_22:1) | 2.239 | 0.0461 | 0.8970 |
| PE(O-22:1_18:1) | -2.235 | 0.0461 | 0.8970 |
| PC(16:0_18:2) | 2.222 | 0.0464 | 0.8970 |
| PI(13:1_18:1) | -2.198 | 0.0471 | 0.8970 |

C. Nominal associations in the fast-versus-slow progression comparison (P < 0.05; n = 15)

| Lipid species | t statistic | Nominal P value | FDR |
| --- | --- | --- | --- |
| PE(17:0_20:4) | 3.019 | 0.0032 | 0.9864 |
| PE(P-18:0_20:4) | 2.528 | 0.0130 | 0.9864 |
| SM(d18:1/16:1) | 2.465 | 0.0154 | 0.9864 |
| Cer(d17:1/25:1) | 2.367 | 0.0198 | 0.9864 |
| SM(d18:1/16:0) | 2.346 | 0.0209 | 0.9864 |
| PE(O-22:1_20:4) | 2.225 | 0.0283 | 0.9864 |
| PC(16:0_18:1) | 2.187 | 0.0310 | 0.9864 |
| SM(d18:2/14:0) | 2.135 | 0.0353 | 0.9864 |
| PG(22:0_16:1) | 2.114 | 0.0370 | 0.9864 |
| PE(O-20:1_20:4) | 2.105 | 0.0378 | 0.9864 |
| PE(O-24:1_20:4) | 2.090 | 0.0391 | 0.9864 |
| Carnitine C10:2 | -2.078 | 0.0403 | 0.9864 |
| PE(O-17:2_20:4) | 2.024 | 0.0458 | 0.9864 |
| TG(18:2_18:3_18:4) | -2.007 | 0.0474 | 0.9864 |
| PG(16:0_20:0) | 2.000 | 0.0481 | 0.9864 |

Notes: Lipid abundances were residualized for age, sex, and BMI and inverse-normal transformed. Section A lists associations that remained significant after Benjamini–Hochberg correction. Sections B and C include only subgroup associations with nominal P < 0.05. Fast and slow progressors were defined by the median baseline ΔFS. None of the subgroup associations survived FDR correction across the 890 tested lipids. β, regression coefficient; ALSFRS-R, Amyotrophic Lateral Sclerosis Functional Rating Scale–Revised; ΔFS, disease progression rate.

**Supplementary Table 3. Sensitivity analyses of the 16 nominal PRS-by-disease-status interactions under alternative clinical covariate adjustments.**

| **Lipid** | **PRS** | **M0: β (SE); P** | **M1: β (SE); P** | **M2: β (SE); P** | **M3: β (SE); P** | **M4: β (SE); P** |
| --- | --- | --- | --- | --- | --- | --- |
| Carnitine C14:0 | BFP | -0.211 (0.064); 0.0011 | -0.211 (0.064); 0.0012 | -0.201 (0.063); 0.0018 | -0.215 (0.064); 0.0010 | -0.201 (0.064); 0.0019 |
| Carnitine C16:1 | BFP | -0.200 (0.068); 0.0038 | -0.198 (0.069); 0.0044 | -0.190 (0.068); 0.0060 | -0.200 (0.069); 0.0043 | -0.189 (0.068); 0.0064 |
| Carnitine C18:3 | WBFFM | -0.217 (0.078); 0.0062 | -0.217 (0.079); 0.0066 | -0.208 (0.077); 0.0076 | -0.218 (0.079); 0.0063 | -0.222 (0.078); 0.0051 |
| Carnitine C8:0 | BFP | -0.159 (0.059); 0.0079 | -0.160 (0.060); 0.0082 | -0.143 (0.056); 0.0117 | -0.161 (0.060); 0.0080 | -0.152 (0.059); 0.0116 |
| Carnitine C10:0 | BFP | -0.150 (0.057); 0.0093 | -0.148 (0.057); 0.0106 | -0.132 (0.054); 0.0154 | -0.151 (0.058); 0.0097 | -0.141 (0.057); 0.0147 |
| Carnitine C12:1 | BFP | -0.151 (0.059); 0.0114 | -0.146 (0.059); 0.0152 | -0.133 (0.058); 0.0220 | -0.148 (0.060); 0.0144 | -0.138 (0.059); 0.0208 |
| Carnitine C20:4 | BW | 0.218 (0.093); 0.0204 | 0.227 (0.093); 0.0161 | 0.215 (0.092); 0.0210 | 0.224 (0.094); 0.0180 | 0.229 (0.094); 0.0154 |
| Carnitine C6:0 | BFP | -0.186 (0.075); 0.0144 | -0.183 (0.076); 0.0165 | -0.171 (0.074); 0.0229 | -0.191 (0.076); 0.0124 | -0.175 (0.076); 0.0219 |
| Carnitine C12:0 | BFP | -0.141 (0.057); 0.0144 | -0.137 (0.057); 0.0183 | -0.124 (0.056); 0.0264 | -0.141 (0.057); 0.0153 | -0.132 (0.057); 0.0227 |
| Carnitine C20:3 | BW | 0.214 (0.095); 0.0257 | 0.221 (0.096); 0.0220 | 0.207 (0.094); 0.0291 | 0.223 (0.096); 0.0215 | 0.218 (0.096); 0.0241 |
| Carnitine C14-OH | BFP | -0.178 (0.076); 0.0208 | -0.177 (0.077); 0.0228 | -0.166 (0.076); 0.0305 | -0.180 (0.077); 0.0213 | -0.162 (0.076); 0.0339 |
| Carnitine C14:1 | BFP | -0.155 (0.064); 0.0174 | -0.146 (0.064); 0.0252 | -0.134 (0.063); 0.0352 | -0.146 (0.065); 0.0261 | -0.140 (0.065); 0.0319 |
| Carnitine C18:0 | BFP | -0.156 (0.073); 0.0328 | -0.159 (0.073); 0.0305 | -0.153 (0.073); 0.0373 | -0.163 (0.073); 0.0280 | -0.149 (0.073); 0.0415 |
| SM(d18:1/23:0) | BW | -0.227 (0.109); 0.0396 | -0.229 (0.110); 0.0392 | -0.236 (0.110); 0.0335 | -0.240 (0.109); 0.0290 | -0.234 (0.110); 0.0347 |
| PC(16:0_16:0) | ALM | -0.117 (0.059); 0.0479 | -0.117 (0.059); 0.0489 | -0.113 (0.059); 0.0583 | -0.114 (0.059); 0.0534 | -0.122 (0.059); 0.0421 |
| TG(16:0_16:1_18:1) | WBFFM | 0.176 (0.088); 0.0478 | 0.175 (0.088); 0.0492 | 0.174 (0.089); 0.0513 | 0.176 (0.089); 0.0492 | 0.173 (0.088); 0.0528 |

Values are interaction coefficients β (standard error); nominal P value. M0: original model including PRS, disease status, PRS × disease status, age, and sex. M1: M0 + BMI. M2: M0 + disease duration. M3: M0 + ΔFS. M4: M0 + BMI + disease duration + ΔFS. Disease duration and ΔFS were coded as zero in controls to model additional variation within the ALS group. Fifteen of 16 interactions remained nominally significant in M4; none of the individual interactions survived correction for multiple testing in the primary analysis. BW, birth weight; BFP, body fat percentage; ALM, appendicular lean mass; WBFFM, whole-body fat-free mass.
